# Patient-derived head and neck tumor slice cultures: a versatile tool to study oncolytic virus action

**DOI:** 10.1038/s41598-022-19555-0

**Published:** 2022-09-12

**Authors:** Annette Runge, Melissa Mayr, Theresa Schwaiger, Susanne Sprung, Paolo Chetta, Timo Gottfried, Jozsef Dudas, Maria C. Greier, Marlies C. Glatz, Johannes Haybaeck, Knut Elbers, Herbert Riechelmann, Patrik Erlmann, Monika Petersson

**Affiliations:** 1 Department of Otorhinolaryngology, Head and Neck Surgery, Medical University of Innsbruck, Innsbruck, Austria; 2 ViraTherapeutics GmbH, Rum, Austria; 3 Institute of Pathology, Neuropathology and Molecular Pathology, Medical University of Innsbruck, Innsbruck, Austria; 4 Boehringer Ingelheim RCV GmbH & Co KG, Vienna, Austria; 5 Diagnostic and Research Center for Molecular BioMedicine, Institute of Pathology, Medical University Graz, Graz, Austria

**Keywords:** Cancer models, Head and neck cancer, Tumour heterogeneity, Tumour immunology, Immunotherapy, Infection, Translational immunology, Tumour immunology, Cancer, Immunology, Oncology

## Abstract

Head and neck cancer etiology and architecture is quite diverse and complex, impeding the prediction whether a patient could respond to a particular cancer immunotherapy or combination treatment. A concomitantly arising caveat is obviously the translation from pre-clinical, cell based in vitro systems as well as syngeneic murine tumor models towards the heterogeneous architecture of the human tumor ecosystems. To bridge this gap, we have established and employed a patient-derived HNSCC (head and neck squamous cell carcinoma) slice culturing system to assess immunomodulatory effects as well as permissivity and oncolytic virus (OV) action. The heterogeneous contexture of the human tumor ecosystem including tumor cells, cancer-associated fibroblasts and immune cells was preserved in our HNSCC slice culturing approach. Importantly, the immune cell compartment remained to be functional and cytotoxic T-cells could be activated by immunostimulatory antibodies. In addition, we uncovered that a high proportion of the patient-derived HNSCC slice cultures were susceptible to the OV VSV-GP. More specifically, VSV-GP infects a broad spectrum of tumor-associated lineages including epithelial and stromal cells and can induce apoptosis. In sum, this human tumor ex vivo platform might complement pre-clinical studies to eventually propel cancer immune-related drug discovery and ease the translation to the clinics.

## Introduction

Head and Neck Squamous cell carcinoma (HNSCC) is the seventh most common malignancy in the world, with an incidence of about 8/100,000 and 387,117 deaths worldwide in 2020^1^. HNSCC tumors commonly arise from the oral cavity, larynx and pharynx. In addition, there is an increasing incidence of oropharyngeal HNSCC attributed to oncogenic Human Papilloma Virus (HPV) infection^2^. The complex anatomy and distinguished etiology are linked to a multitude of molecular changes and mutations, thereby causing genomic instability and propelling cancer progression^3^. Treatment at incident diagnosis usually comprises surgical resection with adjuvant radiation or chemoradiation therapy (CTR)^2^. If complete resection is not feasible, radiation with or without concurrent chemotherapy can be an up-front therapy with curative intention^4^. In PD-L1 negative HNSCCs, combination treatment with the EGFR monoclonal antibody cetuximab and radiation constitutes an additional first line therapeutic option^5^. Unfortunately, 60% of the patients first present with advanced local disease and regional lymph node involvement, which carries a high risk of recurrence (15–40%) and distant metastasis^2^. Recently, immune therapy with a-PD-1 blockage demonstrated promising results in recurrent and metastatic disease. However, progression free survival (PFS) lasting more than 6–9 months has occurred only in 20–23% of the cases^6^. This poor response might partially be due to an immunosuppressive tumor microenvironment (TME) in HNSCC^7^. Therefore, additional therapeutic approaches that enhance the attraction of immune cells by creating a proinflammatory milieu are urgently needed. In this line, oncolytic viruses (OVs) might constitute powerful combination partners. To this end, several OVs are subjected to clinical trials—as monotherapies or in combination with checkpoint inhibitors or other immunomodulatory drugs^8–10^. OVs are biologically active and exert different modes of action to eradicate the tumor bulk. While healthy cells with intact anti-viral innate immune response clear the OV mostly via the type I interferon (IFN) system, the OV is capable to propagate and lyse tumor tissues with a deficient innate immune milieu. OVs not only directly infect and kill tumor cells (oncolysis), but can also boost immune-stimulatory responses. Consequently, activated immune cells could recognize, infiltrate and attack the tumor.

However, due to the heterogeneity between individual HN tumor ecosystems, the prediction whether a particular combination treatment will eradicate HNSCCs and prolong survival remains challenging. This highlights the importance of more translational 3D surrogate systems preserving the individual polyclonal nature of a human tumor ecosystem including the tumor microenvironment (i.e. immune cells, stromal compartments). Different ex vivo culturing approaches have been described with different levels of complexity, e.g. spheroids, organoids or precision cut slice cultures^11^. In this regard, several lines of evidence showed that short term cultivation of HNSCC slices is per se feasible and this technology is well suited to study effects of common therapeutics e.g. Cisplatin, 5′FU, cetuximab or radiation^12–14^. Most frequently used read-outs comprised proliferation and viability assays and detailed histopathological analysis including IHC/IF^15^.

Here, we sought to establish and characterize a patient-derived HNSCC slice culturing system to not only preserve the tumor and stromal architecture but also the immune cell compartment. We reasoned whether T-cells are preserved, functional and could be activated. This could eventually help to evolve such a patient-derived slice culture system to study immediate cancer-immunomodulatory drug actions within the HN tumor ecosystems. Next, we aimed to assess the potential of the OV VSV-GP to infect and propagate within patient-derived HNSCC tumor ecosystems. VSV-GP is a genetically modified Vesicular Stomatitis Virus (VSV) with an exchanged glycoprotein (GP) derived from LCMV (lymphocytic choriomeningitis virus)^16^. Oncolytic and immuno-stimulatory activities as well as the enhanced safety profile of VSV-GP have been demonstrated in vitro and in vivo^17–19^. To date, permissivity and mode of action of VSV-GP in a more translational setting remains to be elucidated.

## Results

### Patient-derived HNSCC slices can be cultured ex vivo

**Figure 1:**
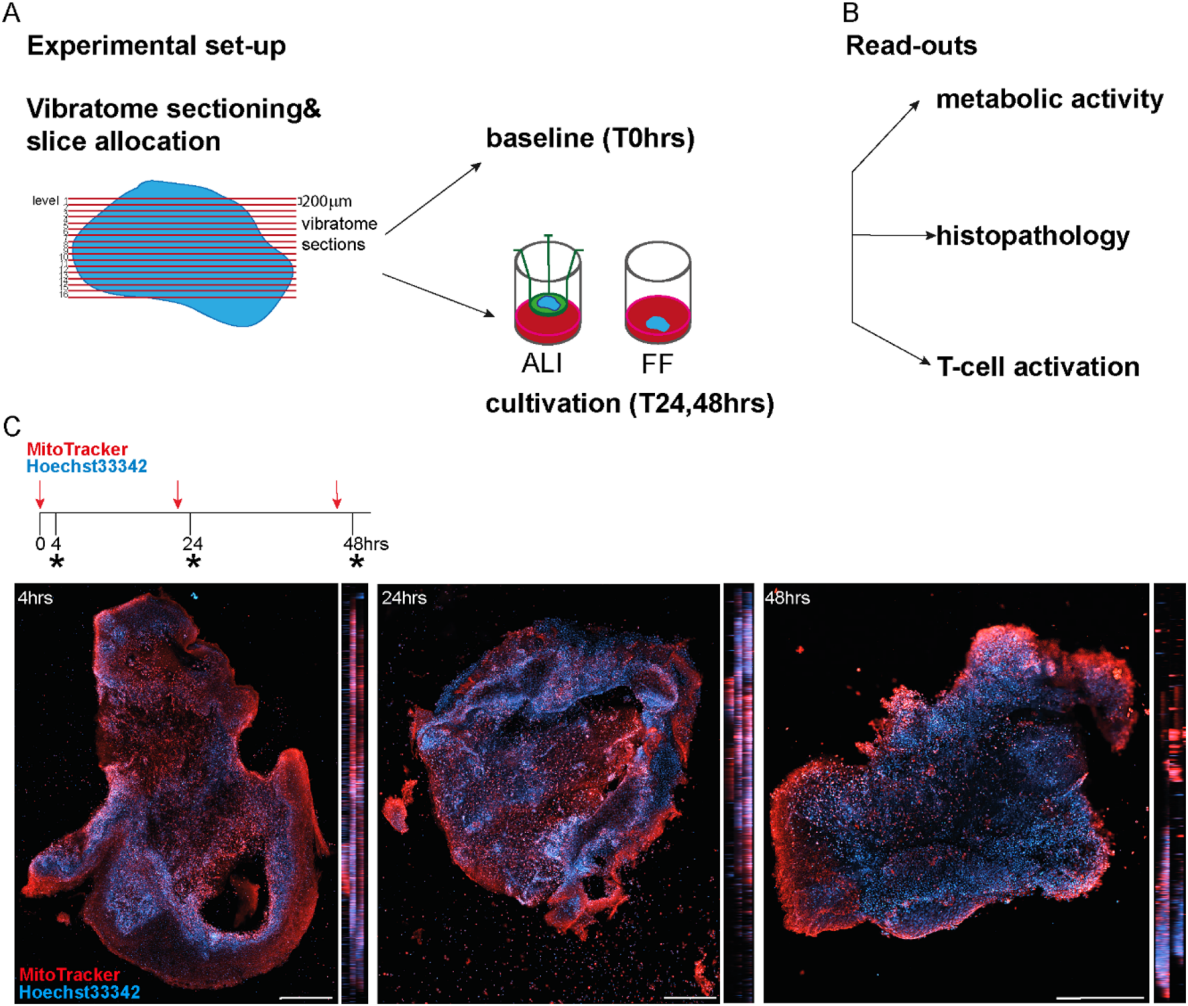
Experimental set-up for patient-derived HNSCC slice cultures and live assessment of metabolic activity. (**A**) Fresh HNSCC biospecimens were sectioned into 200 μm slices using a vibratome. While baseline samples were directly preserved after sectioning, remaining slices were cultivated using air–liquid interface (ALI) and/or free-floating (FF) conditions. (**B**) HNSCC slices were analyzed at 24 and 48 h post culturing and compared to baseline samples (0 h). Metabolic activity was assessed via live confocal microscopy and histopathological evaluations were performed to analyze tumor content, viability and necrosis (H/E staining). Subsequent characterization of the HNSCCs ex vivo cultures was conducted by immunofluorescence studies analyzing tumor, stromal and immune cell compartments. To investigate whether T-cells were still functional, IFNγ secretion in the supernatant of free-floating (FF) slice cultures was determined upon stimulation. Concomitantly, cultured samples were subjected to flow cytometric analysis. (**C**) To measure metabolic activity at 4, 24 and 48 h post culturing, MitoTracker (red), a marker labeling active Mitochondria, was added to the ALI cultures 4 h prior to live confocal microscopy. Hoechst 33342 (blue) was used to stain nuclei. XY and YZ projections are displayed. Scale bars represent 500 µm.

Following sample acquisition and immediate transport to the laboratory, fresh HNSCC biospecimens were sectioned using a vibratome. Tissue slices were preserved just after sectioning (baseline sample) and submitted for pathological evaluation (Fig. 1A). Only samples with histologic features suggestive of high-grade squamous intraepithelial lesion (SIL) or invasive SCC were evaluated as adequate, retrospectively. Details on clinical and histopathological parameters of analyzed cases are reported in Suppl Table 1. Vibratome slices from different levels of the biospecimen were directly cultured for 24 and 48 h either in an air–liquid (ALI) or free-floating (FF) environment*.* An air–liquid approach offers an optimal gas exchange and support with nutrients from the medium and is thought to mimic the original tumor milieu^20^. Of note, ALI approach was utilized to study oncolytic virus actions. Within the free-floating culture, the specimen is fully surrounded by nutrients and medium, cytokines growth factors produced by the tissue are more homogeneously distributed and therefore is well suited to study immunomodulatory actions. In the course of the culturing period, metabolic activity was assessed via confocal live microscopy and T-cell function was tested. In addition, cultured samples were submitted for pathological evaluation by means of H&E and immunofluorescence studies were performed (Fig. 1B).

To establish a patient-derived slice culturing system for HNSCC, we tested tissue chopping versus vibratome sectioning (data not shown), and air–liquid versus free-floating conditions. No supplements such as growth factors or serum were added to the medium. Three slices (200 μm thickness) from different layers of the biospecimen were included in each group and tissue was harvested at 48 h. Histopathological analysis revealed that cytology and tissue architecture were preserved in both air–liquid and free-floating conditions, despite minor intra- and inter-patient variations (Suppl Fig. S1, n = 2 HNSCC patients).

**Figure 2:**
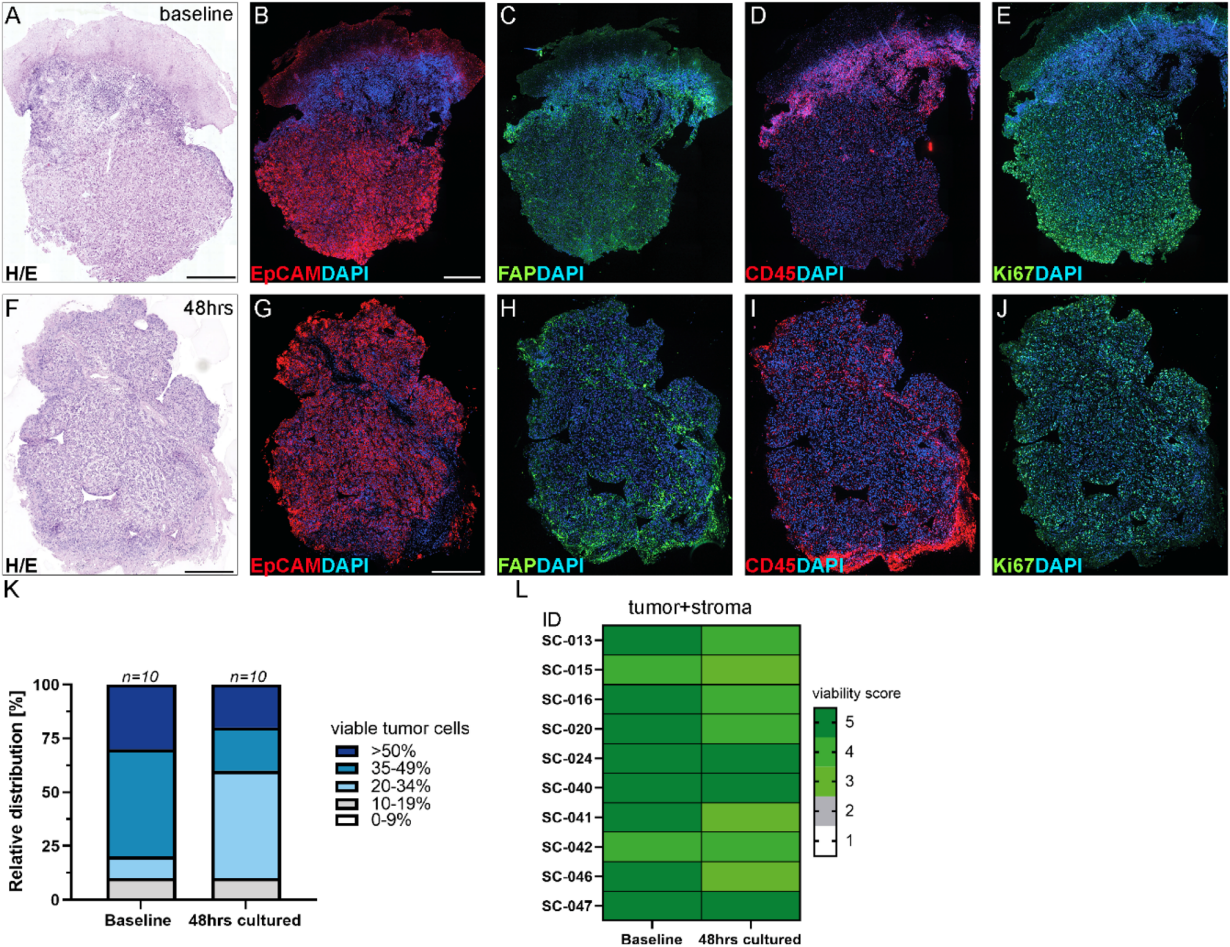
Patient-derived HNSCC slice cultures retain tissue composition ex vivo. (**A**,**F**) Morphology of baseline (0 h) and ALI cultured (48 h) HNSCC slices (SC-024) was assessed (H/E). Immunofluorescent stainings were performed on baseline (**B**–**E**) and 48 h cultures (**G**–**J**) to analyze the presence of the tumor tissue (**B**,**G**) (EpCAM-red), the stromal compartment (**C**,**H**) (FAP-green) and the immune compartment (**D**,**I**) (CD45-red). Proliferation was assessed in baseline and 48 h cultures (**E**,**J**) (Ki67-green). DAPI (blue) was used as nuclear counterstain. Scale bars represent 200 µm. (**K**,**L**) Relative distribution and viability of baseline and cultured patient derived HNSCC slices was determined via digital pathology (n = 10 HNSCCs). (**K**) Graph depicts relative distribution according to percentages of viable tumor cells. (**L**) A 5-tiered scoring system was used to classify the percentage of viable cells within the tumor and stroma: 1 for less than 10%, 2 for 10–34%, 3 for 35–50%, 4 for 51–80% and 5 for more than 80%. Cultured and respective baseline specimens displayed a viability score ranging from 3–5.

Next, we sought to quantify viable and necrotic areas within the baseline and cultured samples using digital pathology on histological sections (Fig. 2A,F, Suppl. S2A,F).

First, we investigated the relative distribution of viability of pooled 48 h cultured versus freshly sectioned baseline slices (n = 10 cases (Fig. 2K)). The majority of the tumor tissue remained viable, albeit a slight shift in the relative distribution was observed in the cultured specimens. In general, necrosis in the cultured tumor slices was detected on the edges and less in the core of the samples.

To directly compare viability of the whole tumor ecosystem pre- and post-culture, a five-tiered scoring system was utilized for subsequent classification (Fig. 2L, adapted from Gavert et al.^21^: 1 for less than 10%, 2 for 10–34%, 3 for 35–50%, 4 for 51–80% and 5 for more than 80% viable cells). Interestingly, 40% of the cultured samples shared same viability scores compared to the respective baselines and in 40%, only slight alterations were detected. In 20% of the samples, a shift of two levels was seen (n = 10 HNSCCs).

This suggests that the success rate of the employed culturing approach was around 80%.

Subsequent experiments focused on a time interval of 48 h.

In sum, HNSCCs from different locations, with different degrees of disease progression or tissue composition could be cultured ex vivo.

Metabolic activity was assayed at different time points by using the MitoTracker dye, which marks metabolic active mitochondria. Four hours prior to analysis, MitoTracker was added to the culture and confocal live cell imaging with a concomitant nuclear counterstain was performed at 4, 24 and 48 h post air–liquid culturing (Fig. 1C). At all time points, the majority of the cultured HNSCC slice encompassed MitoTracker positive (+ve) cells (n = 2 HNSCCs, representative XY and YZ projections of one case are shown), although a slight drop was observed at 48 h. This indicates that the cultured patient-derived tumor slice cultures still retained metabolically active.

To investigate the morphology and composition of the cultured HNSCC tumor ecosystems in more detail, immunofluorescence studies were performed (Fig. 2, Suppl Fig. S2). Specifically, the tumor epithelium (EpCAM), stromal compartment (FAP), immune cells (CD45) and proliferation (Ki67) were analyzed in baseline (Fig. 2B–E, Suppl Fig. S2B–E) and 48 h after culturing (Fig. 2G–J, Suppl Fig. S2G–J; representative images from two cases are displayed). The EpCAM+ve tumor cell compartment represented the majority of both baseline and cultured HNSCC samples. HNSCC slice cultures showed comparable levels of EpCAM at 48 h (Fig. 2B,G, Suppl Fig. S2B,G). In addition, FAP+ve stromal cells were abundant in the stromal part and a similar staining pattern of FAP was observed at baseline versus 48 h cultured HNSCC slices (Fig. 2C,H, Suppl Fig. S2C,H). While in some cases, hyperplastic, strongly infiltrated tissue was observed in the baseline, this benign tissue was mainly abrogated after culturing. The immune cells (CD45+ve) were interspersed in the stromal compartment, but could barely be detected within the tumor epithelium in both, baseline and 48 h cultured HNSCC slices (Fig. 2D,I, Suppl Fig. S2D,I).

Of note, proliferation was detected in baseline and cultured samples, although an intra- and inter-patient variation in of Ki67+ve cells was apparent (Fig. 2E,J, Suppl Fig. S2E,J).

Taken together, the morphology and cellular integrity of patient-derived HNSCC slice cultures was preserved over a time period of 48 h (n = 10 patient-derived HNSCC slice cultures). Importantly, patient-derived HNSCC slice cultures retained not only the tumor and stromal components but also the immune cell compartment.

### T-cells are functional in human HNSCCs ex vivo cultures

**Figure 3:**
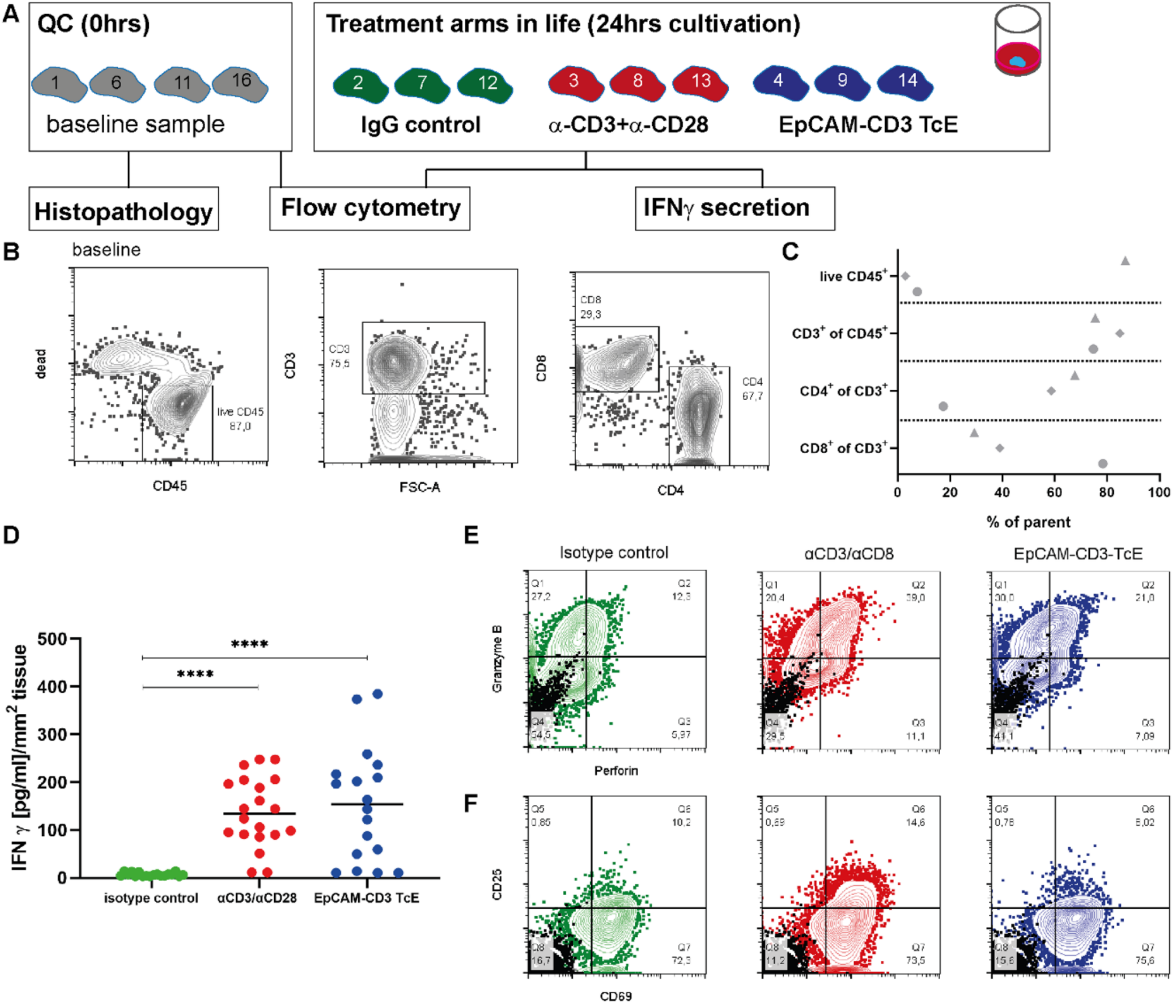
T-cells are functional and can be stimulated in HNSCCs ex vivo. (**A**) Following vibratome sectioning (200 μm) of fresh human HNSCC biospecimens, three slices from different layers were allocated to different treatment arms under free-floating conditions. Baseline samples from different levels were preserved for histopathological analyses. To stimulate T-cell activation, slices were incubated either with a combination of α-CD3/α-CD28 antibodies or with a bispecific T-cell engager (EpCAM-CD3 TcE). A treatment arm with unspecific IgG antibodies served as negative control. Flow cytometry was performed to analyze the immune cell content at baseline as well as effector and activation markers following treatments. IFNg secretion was measured in the supernatants of the different treatment arms. (**B**) Immune cell content at baseline was measured via flow cytometry (n = 3 HNSCCs). An exemplary flow cytometry plot of a representative baseline tumor sample (SC-090) is displayed. Frequency of living CD45+ve immune cells was identified in the single cell population and subsequently CD3+ve cells were gated. CD3+ve cell population was further divided into CD8 and CD4 expressing cells (detailed gating strategy see Suppl Fig. S3). The respective populations (displayed as % parental) were plotted in (**C**). Immune cell content of living CD45+ves (percentage of single cells of interest) varied between three analyzed HNSCCs (circle-SC084, rectangle-SC-087, triangle-SC-090), but the content of CD3+ves was quite similar. CD3+ve population contained both, CD4+ve and CD8+ve cells. (**D**) IFNγ secretion in the different treatment groups was normalized to the area of the slice cultures (μm^2^). IFNγ in the supernatants was significantly increased either following α-CD3/α-CD28 or EpCAM-CD3 TcE treatment (n = 3 HNSCCs). IFNγ secretion was measured in technical duplicates. Student’s *t* test was performed, ****p < 0.0001. Time point of analysis was 24 h post treatment. (**E**,**F**) Flow cytometric analysis of T-cell (CD8) activation molecules (CD25, CD69) and effector molecules (Granzyme B, Perforin) at 24 h. An example of a representative tumor sample (SC-090) is displayed. The quadrant gate was drawn using samples stained with isotype control antibodies conjugated with the respective fluorochromes (black) to exclude unspecific background. In comparison to the isotype-treated control samples, Perforin/Granzyme B double positive populations were increased after α-CD3/α-CD28 or EpCAM-CD3 TcE stimulation. CD69/CD25 double positive populations were slightly increased upon stimulation. Technical triplicates were pooled.

Next, we sought to test whether T-cells were still functional and could be stimulated in patient-derived HNSCC slice cultures (Fig. 3). Following sectioning, three slices from different layers of the tumor sample were assigned to different groups. Again, baseline samples were taken for a histopathological assessment and biospecimens with a tumor content of more than 70% and less than 30% necrosis were included in this analysis, retrospectively (Suppl Fig. S3J–M, n = 3 HNSCCs). In parallel, immune cell content was analyzed at baseline via flow cytometry (Fig. 3C, Suppl Fig. S3A–G). The majority of CD45+ve immune cells were CD3+ve. The CD3+ve population comprised both, cytotoxic CD8+ve and regulatory CD4+ve cells (Fig. 3B). It is important to note, that the immune cell content (CD45+ves) was variable between different tumor patients at baseline (Fig. 3C, n = 3HNSCCs).

To stimulate T-cell activation, one group was treated with a combination of human α-CD3 and human α-CD28 antibodies. A second treatment arm was incubated with a bispecific EpCAM-CD3 T-cell engager. IgG-treated cultures served as negative controls. IFNg secretion in the supernatant was measured 24 h following treatment. To facilitate sampling of the supernatant, a free-floating approach was used. A normalization of IFNg levels to the area of the slices was performed. The treatment with human α-CD3 and α-CD28 as well as with the bispecific EpCAM-CD3 T-cell engager resulted in a significant increase in IFNγ secretion in the supernatant (Fig. 3D, n = 3HNSCCs, technical triplicates). In contrast, little to no IFNγ levels could be detected in the IgG-treated controls (Fig. 3D). Parallel flow cytometric analysis revealed an increase of T-cells (CD8+ve) positive for effector molecules (Granzyme+ve/Perforin+ve) at 24 h post stimulation in comparison to the controls (Fig. 3E, Suppl Fig. S3H). A slight increase in activation molecules within the T-cell CD8+ve population either in the α-CD3/α-CD28 or in the EpCAM CD3 T-cell engager treatment arm was observed (Fig. 3F, Suppl Fig. S3I, n = 2/3 HNSCCs).

These results indicate that the T-cells were still active and could be stimulated in these patient-derived HNSCC tumor ecosystems. Moreover, the data suggest that patient-derived HNSCC slice cultures could constitute a valuable preclinical tool to propel cancer immune-related drug discovery.

### Patient-derived HNSCC slice cultures are susceptible to the oncolytic virus VSV-GP

Next, we sought to investigate whether patient-derived HNSCC slice cultures were susceptible to oncolytic virus infection and therefore could be employed to study oncolytic virus action. Here, we used the oncolytic virus VSV-GP-GFP, a modified Vesicular Stomatitis Virus with an enhanced safety profile, concomitantly equipped with a GFP-tag for visualization^17^. This GFP-tag allows following virus propagation and spread via live microscopy and therefore serves as a read out for permissivity. Permissivity ascribes the potency of a virus not only to infect the tissue but also to produce infectious progeny being able to infect other neighboring cells.

To establish a robust permissivity scoring, we first used murine tumor slice cultures derived from a highly permissive syngeneic, monoclonal CT26.Cl25 IFNAR^−/−^ model were the type I IFN α receptor is depleted (unpublished data) and a semi-permissive TC1 model^22^ (Suppl Fig. S4). We followed input and progeny virus (= GFP+ve signal) at 24 and 48 h post infection (hpi) and performed qPCR on genomic VSV-N copies from the same sample set. Mock (vehicle) and UV-inactivated VSV-GP treated murine tumor slices served as controls. After an infection period of 24 and 48 h, tissue integrity and presence of the GFP signal within each single slice of the respective group was analyzed. A five-tiered system was utilized to score for permissivity: 1-single GFP+ve cells or one small positive patch (< 5 cells), 2-positive patch (around 20 cells) and few interspersed positive cells, 3-huge positive cluster (> 50 cells), 4-multiple positive clusters/big patch and a lot of GFP+ve cells, 5-entire slice encompassing GFP signal. In the CT26.Cl25 IFNAR^−/−^ tumor slice cultures, we observed a rapid increase in virus replication related GFP score (ranging from 3–5) as well as in the genomic VSV-N levels (up to 10^10^ genomic VSV-N copies/slice), which saturated at 24hpi. Slices that received a high GFP score displayed a high genomic VSV-N copy number (Suppl Fig. S4A,C). In contrast, OV treated semi-permissive TC1 tumor slices showed a lower GFP score (ranging from 0–3) and lower VSV-N genome levels (up to 10^7^ genomic VSV-N copies/slice), 24 and 48 hpi (Suppl Fig. S4B,D). Of note, murine tumor slices treated with mock or inactivated VSV-GP-GFP did not show any specific GFP signal and genomic VSV-N copy numbers and therefore were at the limit of detection (Suppl Fig. S4).

Thus, both read-outs, the qualitative GFP scoring allowing for live monitoring of virus replication kinetics and the detection of an increase in intra-cellular viral genomes as a quantitative approach are well suited to assess permissivity.

**Figure 4:**
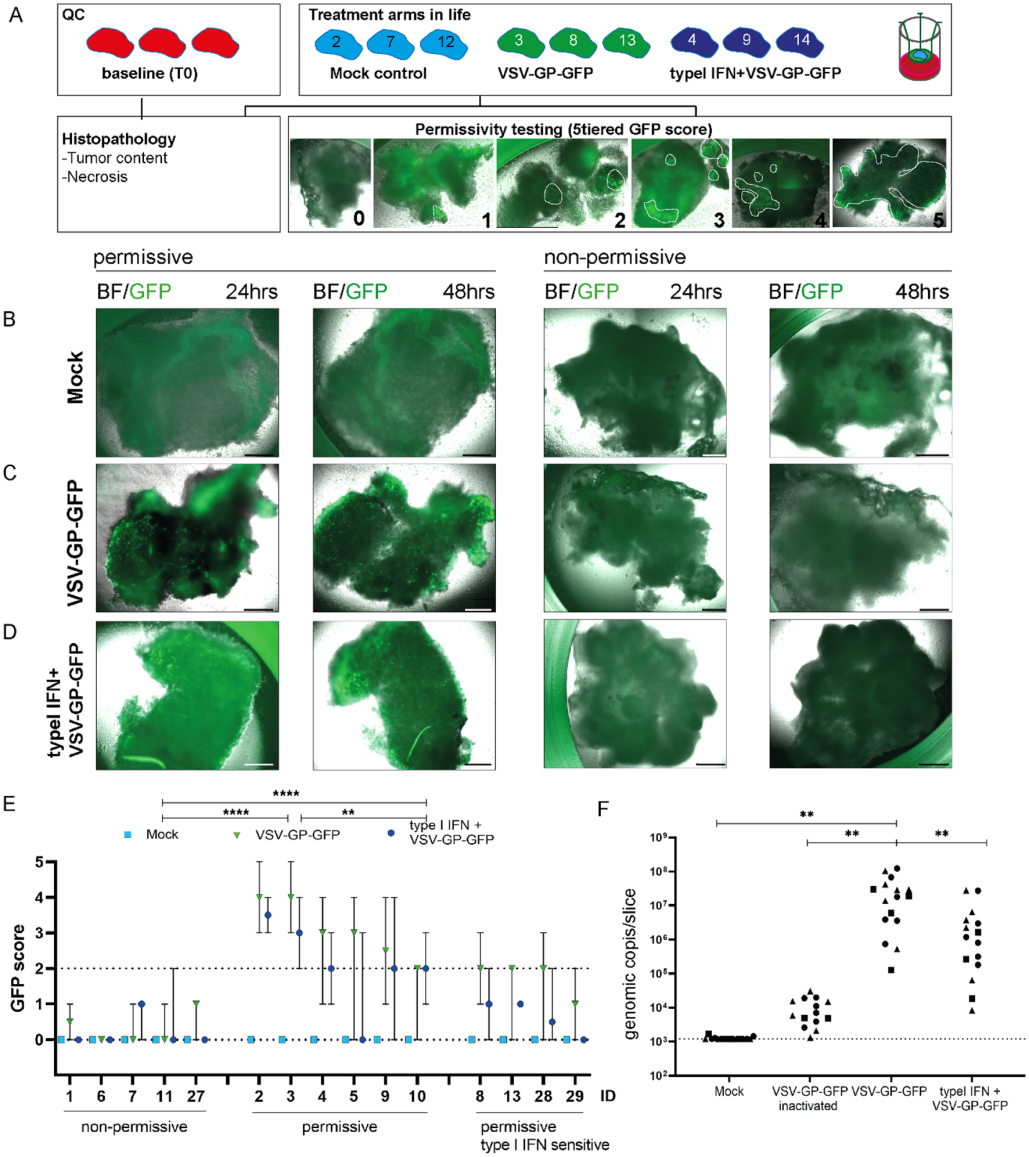
Human HNSCCs are susceptible to VSV-GP ex vivo. (**A**) Following vibratome sectioning of fresh human HNSCC samples, three slices from different layers were allocated to three treatment arms under air–liquid conditions. Baseline samples from different levels were preserved for histopathological analyses. To test for permissivity (production of infectious progeny), slices were treated with a GFP-tagged VSV-GP. In addition, slices were pre-incubated with type I IFN followed by VSV-GP-GFP treatment. Mock treated slices served as controls. Morphology and GFP signal were analyzed via microscopy over time (24 and 48 h). A five-tiered system was implemented to score for permissivity: 1: single GFP+ve cells or one small positive patch (< 5 cells), 2: positive patch (around 20cells) and few interspersed positive cells, 3: huge positive cluster (> 50 cells), 4: multiple positive clusters/big patch and a lot of GFP+ve cells, 5: entire slice encompasses GFP+ve signal. A sample was scored permissive, if the mock treated controls displayed an intact morphology and if 2/3 of the treated slice cultures showed multiple GFP+ve patches (indicated by dashed circles) or an entire slice encompassed GFP (GFP score higher than 2). (**B**–**D**) Live captures of mock (**B**), VSV-GP-GFP (**C**), and type I IFN + VSV-GP-GFP (**D**) 24, 48 h following treatment. Bright field (BF) and GFP (green) pictures of a permissive and non-permissive case are shown. Scale bars represent 500 µm. (**E**) Graph summarizing permissivity assessment (GFP score) of histologically verified HNSCCs (n = 15) at 48 h post treatment. 10/15 patient-derived HNSCC slice cultures received a GFP score of 2 or higher, meaning they were permissive to OV treatment. Type I IFN pre-treatment resulted in a reduction of the GFP signal in 4/10 permissive cases (type I IFN sensitive). 5/15 cases received a score below 2 and were scored non-permissive. Student’s *t* test was performed to determine significance, **p < 0.01 ****p < 0.0001. (**F**) Genomic VSV-N copy number was determined via qPCR using tissue lysates of 48 h cultured samples (n = 3 HNSCCs, square-Patient 27, triangle-Patient 28, circle-Patient 29). An additional treatment arm with inactivated VSV-GP-GFP served as a benchmark for input virus. Genomic copy number was significantly increased 48 h after VSV-GP-GFP treatment compared to mock control and the inactivated virus group. Incubation with type I IFN before VSV-GP-GFP treatment resulted in a significant decrease in VSV-N genomic copies. Genomic copy numbers were measured in technical triplicates with biological quadruplicates for Patient 27 and biological sixtuplicates for Patient 28 and 29. Student’s *t* test was performed to determine significance, **p < 0.01, **** p < 0.0001.

To test for permissivity in the more complex polyclonal patient-derived HNSCC slice cultures, we allocated slices from different layers to different treatment arms. Mock treated slices served as control (Fig. 4A). We used VSV-GP-GFP to follow virus propagation and spread via live microscopy. One pre-requisite for OV action is the deficiency of tumors in type I IFN signaling cascade, a crucial mechanism for anti-viral defenses of the host. To analyze whether type I IFN signaling is the key driver to block OV spread and propagation, we pre-incubated the slices with type I IFN followed by virus treatment in a third treatment arm. 24 and 48 h of cultivation, permissivity indicated by the presence of GFP signal was blindly scored. Only histologically verified cases were included in the study, retrospectively. To score for permissivity, the five-tiered system was employed (Fig. 4A). A HNSCC case was scored as permissive at 48 h, if mock treated controls displayed an intact morphology (Fig. 4B), two out of the three slices displayed multiple GFP+ve patches (consisting of 20 or more cells) or one entire slice encompassed GFP signal (Fig. 4A,C,E). In contrast, HNSCC slice cultures with no GFP signal or a single cluster of GFP+ve cells were scored as non-permissive (Fig. 4A,C,E). All mock treated controls remained negative for GFP signal (Fig. 4B,E). To this end, ten out of fifteen patient-derived HNSCC cultures were scored to be permissive to VSV-GP-GFP. Type I IFN pre-treatment reduced the OV spread in four out of the ten permissive tumors (Fig. 4D,E). Parallel experiments were conducted to quantify virus load in the permissive patient-derived HNSCC slice cultures (n = 3 permissive HNSCCs, Fig. 4F). Besides mock, VSV-GP-GFP and type I IFN treatment arms, we included a group where we incubated HNSCC slice cultures with UV-inactivated VSV-GP-GFP to measure the effect of the input virus.

While mock and inactivated genomic VSV-N copy number was almost similar, a significant increase in viral load in the VSV-GP-GFP permissive HNSCC slice cultures was observed. Interestingly, a pre-incubation with type I IFN prior to OV treatment resulted in a significant decrease in genomic VSV-N copy numbers. Indeed, a certain variation in permissivity levels was observed in both approaches, GFP-scoring and qPCR. Besides intra-tumor heterogeneity, a potential reason for this observation could be the fact that the infection per se is characterized as an stochastic event infecting by chance cells being more or less permissive resulting in cell to cell spread (efficacy of the virus)^23,24^.

From these experiments, we have learned that the oncolytic virus VSV-GP-GFP can infect and propagate in patient-derived HNSCC tumor ecosystems and around 67% (two-thirds) of the analyzed histologically verified human tumor samples were permissive. In addition, pre-incubation with type I IFN reduced viral spread in 40% of the permissive cases.

### Mode of action of VSV-GP in HNSCCs ex vivo

**Figure 5:**
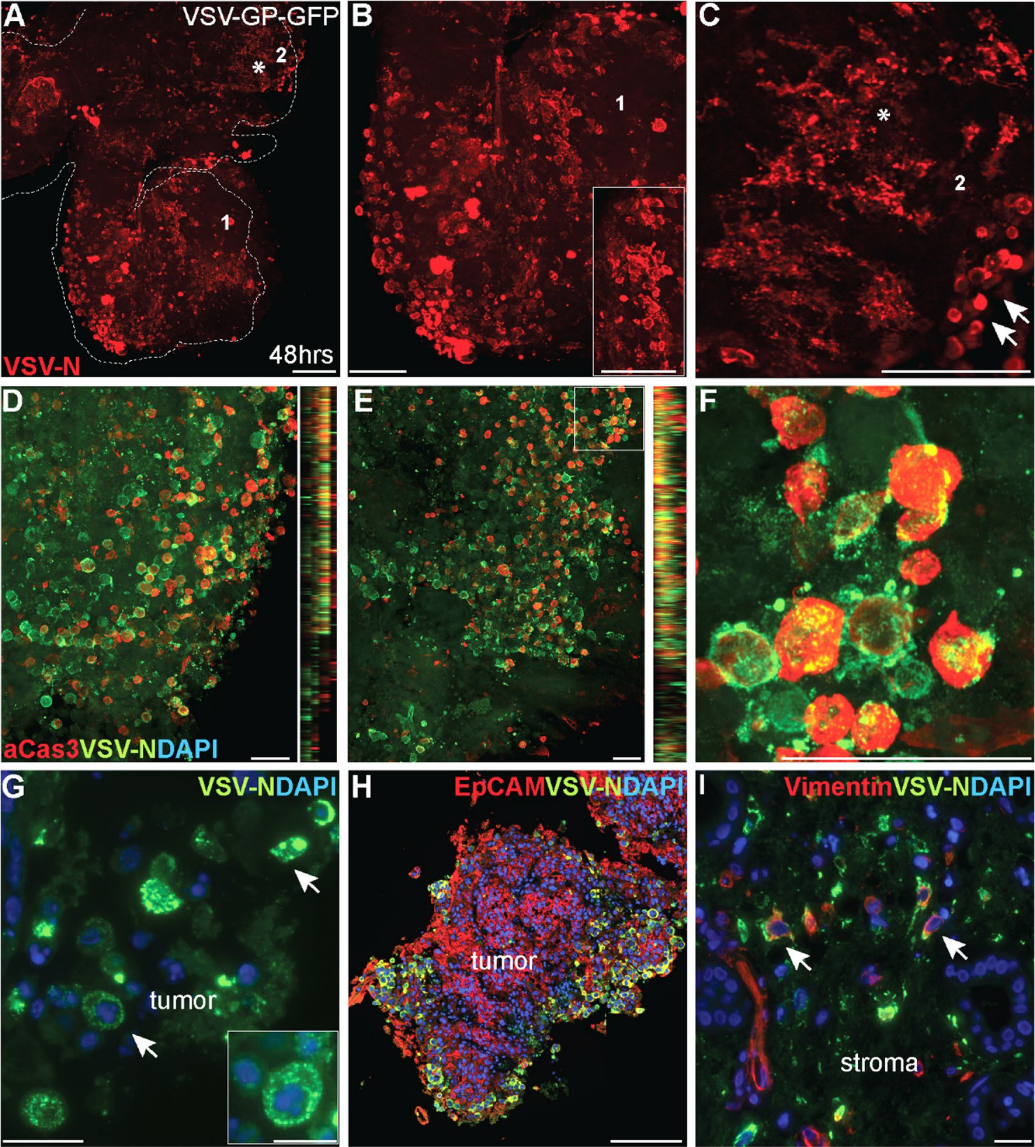
Mode of action of the oncolytic virus VSV-GP in patient-derived HNSCC slice cultures. (**A**–**C**) Whole mount staining using VSV-N antibody (red) on OV treated HNSCC slice cultures at 48 hpi. Samples were cultivated under air–liquid conditions. Virus spread could be monitored throughout the entire slice (**A**). Rounded cells were detected (higher magnification in (**B**) (1), arrows in (**C**) (2) adjacent to virus positive cell debris (**C**, (2), asterisks). Scale bars represent 200 µm. (**D**–**F**) Co-immunofluorescent stainings of VSV-N (green) and aCas3 (red) were performed on two different whole-mounted OV-treated slices 48 hpi. Maximal projections (xy, yz) showed the presence of active Caspase-3 in VSV-N+ve cells (higher magnification in **F**). Scale bars represent 50 µm. VSV-N+ve virus factories (**G**, VSV-N-green) were localized in the cytoplasm of thin sections derived from OV-treated HNSCC slice cultures. Scale bars represent 20 µm and 10 µm in the insert. (**H**,**I**) Virus (VSV-N-green) could be detected in the EpCAM+ve tumor cell compartment (EpCAM-red, **H**). Some VSV-N+ve cells co-localized with Vimentin (red) in the tumor stroma (**I**). DAPI (blue) was used as nuclear counterstain. Scale bars represent 20 µm.

To get a deeper insight into the action of the OV VSV-GP on a cell biological level, we performed immunofluorescence studies (Fig. 5). First, we analyzed the presence and localization of the Nucleoprotein N of VSV-GP throughout the whole-mounted HNSCC slices at 48 hpi. Intriguingly, OV spread could be monitored through the entire patient-derived HNSCC tumor ecosystem. Among the infected cells, a large N+ve proportion comprised rounded disintegrating cells (Fig. 5A–C). These rounded N+ve cells could also be detected adjacent to N+ve cell debris. On a cellular level, N signal was organized in speckles and localized to the cytoplasm (Fig. 5G), which is indicative for virus factories/liquid organelles^25^. Subsequent co-immunofluorescent studies on infected, whole-mounted HNSCC slice cultures demonstrated that a substantial amount of N+ve infected cells also expressed active caspase 3 (aCas3), implying that VSV-GP-GFP can induce apoptosis in patient-derived HNSCC slice cultures (Fig. 5D–F). Of note, not all infected cells showed an aCas3+ve signal.

VSV-N could also be localized within the EpCAM+ve tumor cell compartment, demonstrating that tumor cells can be infected (n = 10/11; Fig. 5H, Suppl Table S1). In the stromal compartment, quite a few N+ve cells were also positive for the mesenchymal marker vimentin, suggesting that VSV-GP could infect cancer-associated fibroblasts (n = 7/9; Fig. 5I, Suppl Table S1).

Thus, these findings infer that the OV VSV-GP could spread throughout the patient-derived tumor ecosystems thereby infecting not only the tumor compartment but also the TME. Along with the infection, VSV-GP-GFP seems to induce apoptosis in a subset of cells.

## Discussion

Over the past decades, tremendous progress has been made in the field of cancer immunotherapy, including a plethora of therapeutic combination options. To date, a predictive tool, which treatment options offer the optimum benefit for the individual patient remains elusive. Accordingly, the bridging from pre-clinical murine tumor models to the complex human tumor ecosystems is crucial. Therefore, we sought to set up a patient-derived HNSCC slice culturing system as a tool to investigate immunomodulatory factors and OV action.

First, the process of sampling and logistics was optimized to avoid deterioration of the biopsied tissue. Concomitantly, we implemented a pathological assessment to verify that the obtained HN biospecimens were viable and indeed contained neoplastic tissue. A reliable short-term cultivation protocol was established, which resulted in preservation of tissue morphology and cellular integrity up to 48 h. No gross alterations were observed with respect to the cultivation approach. Patient-derived HNSCCs could either be cultured using the free floating or the air liquid approach, which is in accordance with published data^14^.

Detailed immunofluorescent studies revealed that the tumor epithelium and the tumor stroma were retained compared to the original in situ contexture. In addition, immune cells were still present in these 3D short term HNSCC ecosystems^26^.

A cultivation period for 3–5 days was per se feasible but resulted in high variations in tissue preservation and viability. Our digital pathology assessment further revealed a success rate of the air–liquid approach of around 80% at 48 h of cultivation. Thus, a time interval up to 48 h was chosen to study effects on viability and permissivity. To expand the time window of cultivation, an addition of supplements e.g. patient-derived extracellular matrix would be useful as previously reported^27,28^. Furthermore, inter-slice variations with regard to the histopathological composition and viability of the biospecimen were observed representing the heterogeneity of each single tumor. Thus, an accurate collection of consecutive slices and representative assignment of slices from different layers was crucial to capture a comprehensive picture for subsequent analyses. Obviously, also inter-patient variability was monitored, pinpointing to the complex etiology and architecture of HNSCCs. This observed heterogeneity is in line with reported findings and probably is inevitable the closer the biological surrogate test system mimics a human tumor ecosystem^26^.

Intriguingly, cytotoxic T-cells were not only present but also functional in patient-derived HNSCC tumor ecosystems. Treatment of HNSCC slice cultures (free-floating approach) with immunostimulatory agents resulted in secretion of significant amounts of IFNγ inferring that immune cells were activated. Furthermore, an increase in cytotoxic T-cells expressing effector molecules could be observed upon treatment. Accordingly, there are some initial reports focusing on other tumor entities, that tumor slice cultures constitute a biological surrogate to unravel the early effects of immunomodulatory cancer agents such as checkpoint inhibitors^29^. With this regard, is tempting to speculate that that the patient-derived HNSCC slice culture system could help to disentangle the complex actions of cancer immunotherapeutics within the HN tumor ecosystems. Obviously, these analyses could only capture immediate intrinsic immune actions within the HN tumor ecosystems and secondary processes of the systemic immune response (such as T-cell priming in the lymph node) are not covered. To overcome these limitations, the rapidly growing field of microfluidic organs-on-chip could be a promising technical asset^30,31^.

Finally, we have demonstrated that patient-derived HNSCC slice cultures (air–liquid approach) constitute a versatile tool to study permissivity and OV action, albeit restricted to the immediate cellular mechanisms within the HN tumor ecosystems. We established a permissivity assay on a cellular as well as genomic level. From these experiments we have learned that the OV VSV-GP-GFP could propagate and spread in a high proportion (67%) of patient-derived HNSCCs ex vivo. In line with previous in vitro findings for VSV-GP, pre-incubation with type I IFN could prevent OV spread inferring a type I IFN dependency of the OV in 40% of the analyzed permissive HNSCC cases^32–35^. The finding that 60% of the permissive cases could not be protected by type I IFN incubation, suggests that these tumor ecosystems were not responsive to type I IFN. Interestingly, a down-regulation of type I IFN signaling is part of the immune evasion strategy and prevention of cell death as tumors evolve^36^.

Interestingly, roughly 33% of the analyzed HNSCC cases were non-permissive. Future studies will unravel whether these cases display alterations in the type I IFN signaling pathway and/or (concomitant) alternate host factors could hamper permissivity. On a cellular level, we have observed that not only tumor cells but also tumor-associated stromal cell lineages could be infected by VSV-GP. In addition, the formation of virus factories was detected in the cytoplasm of infected cells^25^. Infected cells seemed to round up and finally disintegrated. Virus positive cell debris could be detected. Moreover, active caspase 3 was present in a substantial N+ve cell fraction, implying that VSV-GP-GFP induced apoptosis in patient-derived HNSCC slice cultures. This is in accordance with previous reports that VSV-GP or VSV wt primarily induce apoptosis via the activation of effector caspases such as caspase 3^19,32,37,38^.

Active caspase 3 staining was not found in all infected cells, which on the one hand could be a limitation due to protein stability and/or timing. On the other hand, it seems plausible that in some cells the infection could additionally trigger inflammasome activation, which involves the activation of alternate caspases^39^. The complex crosstalk between different cell death pathways triggered by VSV-GP is probably cell-type specific and needs to be addressed in more simple in vitro test systems such as patient-derived organoids.

In sum, the data show for the first time that the OV VSV-GP can infect and propagate in patient-derived HN tumor ecosystems. Together with additional pre-clinical data in vitro and in murine tumor models for VSV-GP, the obtained data within the patient-derived HN tumor ecosystems could facilitate the bridging towards the clinics e.g. patient and/or indication selection^19,32^. To date, tissue explants deprived from distinct tumor entities such as colorectal, breast, prostate, liver and bladder cancer as well as melanoma and glioma have been utilized to study a variety of OV (e.g. HSV, vaccinia virus, NDS, poxvirus, measles)^40,41^. Here, we amend this toolbox to HN cancer and the OV VSV-GP. An alternative to study the potential of OV equipped with immunostimulatory cargos is the use of human tumor-derived ascites mixed with patient-derived PBMCs, which might eventually complement the understanding of OV virus action on an immunological level^42^.

This broad panel of pre-clinical tools of human tumor surrogates can be utilized for initial proof of concept studies and hypothesis generation. In addition, they might eventually provide a guidance to design and potentially accompany clinical studies, as recently demonstrated for an a-PD1 neoadjuvant study^43^.

Unraveling the key determinants why a certain tumor subset is responsive to OV or cancer immunomodulatory drug treatment and another subset of tumors is not susceptible, is one of the most prevailing contemporary issues.

## Materials and methods

### Acquisition of human head and neck cancer biospecimens

Written informed consent of the patients with incident HNSCC was obtained. The study was approved by the ethics committee of the Medical University of Innsbruck, Austria (vote No 1174/2018). All experiments were performed in accordance with relevant guidelines and regulations. The datasets used and/or analysed during the current study available from the corresponding author on reasonable request. Tumor biopsies from the oral cavity, larynx, and pharynx were taken during endoscopy under anesthesia at the Department of Otorhinolaryngology—Head and Neck Surgery, Medical University of Innsbruck, Austria. This diagnostic intervention was part of the initial diagnosis HNSCC. For this study, samples with a diameter of about 5 mm were taken with biopsy forceps from the margins of the primary tumor to provide areas with viable cells. Directly after endoscopy, the biospecimen was placed into M199 medium and cooled.

### Preparation and cultivation of patient-derived HNSCC slices

Tissue biospecimens were transported in M199 medium and kept on ice until sectioning. After removal of connective tissue, the sample was embedded in 1,8% low melting agarose (Biozym #840101) and mounted on a specimen plate using fibrin glue (Surgibond) or cyanoacrylate adhesive (Loctite). Tissue was sectioned into 200 μm slices using a vibratome (LeicaVT1200S, settings: a speed of 0,3 mm/s, 1 mm amplitude, blade angle of 15°). Slices were initially collected in PBS supplemented with 1%Pen/Strep. Directly after sectioning, slices from different layers were preserved for histopathological analysis. The remaining slices were cultured. For the air–liquid approach, slices were placed on tissue culture inserts (Greiner Thincerts, Pore size 0.4 mm) in 24-well plates containing 1:1 medium (one part M199 without phenol red (Gibco #11043-023) and one part KSFM without supplements (Gibco #17005-042)). Subsequently, the slices were covered with additional medium and then cultured in a humidified incubator at 5% CO_2_ and 37 °C. For the free-floating approach, slices were gently transferred into 96-well plates and cultivated under the conditions mentioned above with the addition of 100 µg/ml Primocin (InvivoGen #ant-pm-1). No supplements such as growth factors or serum were added to the medium to preserve the natural environment of these primary 3D tumor ecosystems.

### Preparation and cultivation of murine tumor slices

Animal experiments were performed in compliance with the Austrian experimentation law (animal trial permission granted by the Federal Ministry of Science, Research and Economy BMBWF-66.011/0156-V/3b/2019). Tumors were implanted by subcutaneous injection of 1 × 10^6^ CT26Cl.25 IFNAR^−/−^ cells or 5 × 10^5^ TC1 cells in the right flank of female Balb/c (CT26Cl.25 IFNAR^−/−^) or C57BL/6 (TC1) mice. Tumors were harvested at a size around 0.4 cm^3^ and transported on ice in PBS. Tumor cubes of 0.5–1 mm diameter were prepared and sectioned like human HNSCC biospecimens. The slices were transferred into a 48-well plate and cultured as free-floating approach in 400 µl medium. CT26Cl.25 IFNAR^−/−^ slices were cultured in DMEM media (Gibco #21063-029) and TC1 slices in IMDM (Gibco #21056-023). Both media were supplemented with 1%Pen/Strep (Gibco, #15070063).

### Assessing metabolic activity

To determine metabolic activity at different time points, patient-derived HNSCC slices were cultured under air–liquid conditions in a 24-well plates and Thincerts inserts (Greiner) in 100 μl 1:1 medium. Slice cultures were incubated with MitoTracker Red CMXRos (1 μM, #M7512, ThermoScientific) and Hoechst 33342 (1:2000, H1399, Invitrogen) for 4 h and subsequently imaged using a laser scanning microscope with Airyscan 2 (Cell Discoverer 7, Zeiss). A tile scan of the whole slice was done, and the imaging depth was 250–330 µm (variations due to inclined position) with 19.5 µm intervals.

Slices were analyzed 4, 24 and 48 h following cultivation. XY, YZ Maximum projections of the confocal z-stacks were generated using Zen Blue software.

### Histopathological assessment

Tumor slices were fixed in formalin, washed with phosphate-buffered saline (PBS) and embedded in Histogel (ThermoFisher Scientific #HG-400-012). Samples were then processed for paraffin embedding. Thin sections (4 μm) were cut with a rotating microtome (Thermo Scientific Microm HM 355S) with 2 sections per slide. Slide 1, 9 and 17 were stained with hematoxylin/eosin (HE) and independently assessed by two pathologists. Clinical biopsies of HNSCC may reveal a wide spectrum of pathohistological findings, including normal mucosa, low- or high-grade squamous intraepithelial lesions (SIL) and invasive squamous cell carcinoma (SCC) and necrosis. Only specimens with histologically confirmed high-grade SIL or invasive SCC (WHO Classification of Head and Neck Tumors, 4th edition) were included in the analysis. Because the true nature of the specimen was unknown at the time the biopsy was taken, all samples not complying with the above criteria were retrospectively discarded. Pathologists also estimated the extent of tissue necrosis in the tumor slices before and after 48 h cultivation.

### Establishment of a viability score

To quantify the amount of tumor cells, stromal cells and necrosis in tumor slices at baseline and after 48 h cultivation QUPath 0.3.2 software was used. Cell detection was executed with standard parameters and each cell or cell group was defined as tumor, stromal or necrotic cell. The cell numbers were normalized to the total cell count. The amount of tumor cells together with stromal cells was defined as viable and a 5-tiered scoring system was implemented: 1 for less than 10% viable cells, 2 for 10–34%, 3 for 35–50%, 4 for 51–80% and 5 for more than 81%.

### Immunofluorescence studies

To perform immunofluorescent stainings, paraffin sections were dewaxed in xylene and rehydrated in graded alcohol. For antigen unmasking, sections were retrieved in citrate buffer (BioGenex # HK086-9K) at 121 °C for 20 min using an Antigen Retriever (Aptum Biologics). Following blocking (Dako Antibody Diluent) for 3 h at RT, sections were incubated with primary antibodies at 4 °C o/n (Suppl. Table S3). On the next day, samples washed twice with PBS for 5 min at RT and stained with the appropriate fluorescence-labelled secondary antibody and subsequently with DAPI (0.5 µg/ml, Sigma Aldrich #D9564) for 30 min at RT (Suppl. Table S4). Three washing steps with PBS for 5 min at RT were performed. Slides were rinsed with distilled H_2_O before mounting with Mowiol/DABCO (DABCO: Roth #0718.2; Mowiol-488: Roth #0713.2). For co-immunofluorescent stainings, consecutive stainings were conducted. For whole mount stainings, fixed slices (200 μm) where permeabilized with 0.2% Tween20/PBS for 30 min (all steps were performed on an orbital shaker). Afterwards, the slices were blocked for 3 h with blocking buffer (0.5% milk powder, 0.25% fish skin gelatin, 0.5% Triton X-100 in TBS) and then incubated with the primary antibody o/n at 4 °C (Suppl. Table S3). On the next day, the slices were washed twice with PBS for 15 min and stained with the appropriate fluorescence-labeled secondary antibody for 4 h at RT (Suppl. Table S4). Following counterstaining with DAPI for 15 min, the slices were washed three times for 30 min with PBS. After a last washing step with dH_2_O, the samples were mounted with Mowiol/DABCO^44,45^.

### Immune stimulation and measurement of IFNg secretion

To measure IFNγ secretion upon T-cell stimulation, patient-derived HNSCC slices were cultured under free floating conditions in a 96-well plate in 250 µl 1:1 medium. Three slices from different layers of the biospecimen were allocated to different groups before treatment. One group was incubated with human α-CD3 (1 µg/ml, clone [OKT3], BioLegend) and human α-CD28 (0.5 µg/ml, clone [CD28.2], BioLegend) antibodies, whereas the second group was treated with a bispecific CD3-EpCAM antibody (2 nM^46^). IgG (α-mouse IgG2a (clone [MOPC-173] BioLegend) and α-mouse IgG1 (clone [MOPC-21] BioLegend)) treated triplicates served as negative control. Supernatant of individual slice cultures was analyzed for INFγ secretion 24 h after treatment. The secreted amount of IFNg was measured using an flow cytometry-based bead Assay (Legendplex #740390) according to the manufacturer’s instructions. The area of the respective slice was measured using the region measurement function of the Zen Blue software (Carl Zeiss Microscopy GmbH, Jena). Only slices with a range of 0.9–10 mm^2^ were included in the study. IFNγ secretion (pg/ml) was normalized to the measured areas of the respective tissue slice. The values were plotted in GraphPadPrism and a Student’s *t* test was used to determine significance of the obtained results. p-values < 0.05 were considered significant (*p < 0.05, **p < 0.01, ***p < 0.001).

### Oncolytic virus treatment and permissivity testing

To receive a representative picture of the whole human biospecimens, slices from different layers were allocated to different treatment groups and the respective baseline samples were preserved. All treatment arms comprised triplicates from different layers of the biospecimen. Subsequently, patient-derived HNSCC slices were treated with 1 × 10^7^ TCID_50_ VSV-GP-GFP. VSV-GP-GFP was produced as previously described^17,19^. A second group was pre-incubated with type I IFN (100 U/ml; Pbl assay science #11200-2) for 1 h at 37 °C, 5 °C CO_2_ prior to VSV-GP-GFP treatment. Mock treated slices served as negative control. Samples were cultured at 37 °C, 5% CO_2_ in a humidified incubator. After an infection period of 24 and 48 h, tissue integrity and presence of the GFP signal within each single slice of the respective group was analyzed using a fluorescence microscope (Cell Observer, Zeiss). A five-tiered system was utilized to score for permissivity: 1-single GFP+ve cells or one small positive patch (< 5 cells), 2-positive patch (around 20 cells) and few interspersed positive cells, 3-huge positive cluster (> 50 cells), 4-multiple positive clusters/big patch and a lot of GFP+ve cells, 5-entire slice encompasses GFP+ve signal. Only cases displaying an intact morphology in the mock treated controls were included. In addition, a retrospective histopathological assessment was performed by two independent pathologists. A case was called permissive (= production of infectious progeny = GFP+ve) at 48 h post culturing, if two of the triplicates received a score of two (if more than 3 slices than half of them) or an entire slice encompassed GFP (GFP score higher than 2). At 48 h, samples were fixed and further processed for paraffin embedding.

### Detection of genomic virus copies

To measure genomic VSV-N copies in human and murine tumor slices, slices were treated with 1 × 10^7^ TCID_50_ VSV-GP-GFP. An additional group with 1 × 10^7^ TCID_50_ inactivated VSV-GP-GFP was added as a control for remaining input virus. Inactivation was performed using a UV crosslinker (Boeckel) for 5 min (according to the manufacturer′s instructions). Mock treated slices served as a negative control. For the human samples, a type I IFN pre-incubation group was added. Samples were cultured at 37 °C, 5% CO_2_ in a humidified incubator. The presence of GFP positive cells was analyzed in all slices 24 h and 48 h post infection (hpi). The human slices were harvested after 48 h and placed in RNAlater (Quiagen, #1018087) at 4 °C. The murine slices were harvested 24 and 48 hpi.

RNAlater preserved slices were transferred in a Lysis tube E (Analytic jena, #31-00600) containing 300 µl RLT buffer (Quiagen, #79216) + 1% β-Mercaptoethanol (Sigma, #M6250). Lysis was done in continuous mode for 1 min using a SpeedMill plus (Analytik Jena). Afterwards the cell suspension was centrifuged at 1000*g* for 1 min at 4 °C and the supernatant was transferred into a fresh tube. The RNA was extracted using the MagMax-96 Viral RNA Isolation Kit (ThermoFisher #AM1836) according to manufacturer’s instructions. Genomic VSV-N copies were measured using iTaq Universal Probes One-Step Kit (BioRad, #1725141) with VSV-N primers (forward: 5′-AGTACCGGAGGATTGACGACTAAT-3′, reverse: 5′-TCAAACCATCCGAGCCATTC-3′) and probe (5′-ACCGCCACAAGGCAGAGATGTGGT-3′). Amplification protocol: 50 °C for 10 min, 95 °C for 2 min and 40 cycles of 95 °C 15 s and 60 °C 30 s. A standard curve was set up by using VSV RNA in a concentration from 10^7^ to 10^1^ copies/ml. All qPCR samples were measured in technical triplicates. The values were plotted in GraphPadPrism and a Student’s *t* test was used to determine significance of the obtained results. p-values < 0.05 were considered significant (*p < 0.05, **p < 0.01).

### Flow cytometric analysis

For characterization of infiltrating leukocyte populations two to three tumor slices were transferred onto a prewetted 70 µm cell strainer (Miltenyi Biotec) and mechanically dissociated with the rubberized part of a 3 ml syringe. After the cells have been rinsed through with 10 ml PBS, they were pelleted, and the supernatant was discarded. Single cell suspensions were stained with a live/dead discriminating dye (Zombie Aqua™ Fixable Viability Kit). After blocking human Fc receptors with Human TruStain FcX™, cells were stained extracellular with the following antibody cocktail: mouse α-human CD3 BV650 (clone OKT3), mouse α-human CD45 FITC (clone HI30), mouse α-human CD8α PerCP-Cy5.5 (clone RPA-T8), mouse α-human CD4 PE-Cy7 (clone OKT4), mouse α-human CD25 AlexaFlour700 (clone BC96) and mouse α-human CD69 APC/Fire700 (clone FN50). After fixation and permeabilization (True-Nuclear™ Transcription Factor Buffer Set) and another Fc receptors blocking step, cells were stained with the following antibody cocktail against intracellular proteins: rat α-human Granzyme B BV421 (clone QA18A28), mouse α-human Perforin PE (clone dG9) and mouse α-human FoxP3 AlexaFluor647 (clone 150D). All antibodies and reagents used for staining were purchased from Biolegend. Samples were measured on a MACSQuant16 (Miltenyi Biotec), analyzed using FlowJo (Tree Star Inc) and displayed with GraphPad Prism.

### Microscopy/image processing

H&E stained slides were scanned using the Pannoramic Scan II (3D Histech) and pictures were exported using the Pannoramic Viewer Software. Immunofluorescence stainings were captured using an inverted fluorescence microscope (Cell Observer, Zeiss) (Figs. 2, 5) or Pannoramic Scan II (Suppl. Fig. 2). For the immunofluorescence staining of HNSCC whole-mount slices (200 μm), tile scans and z-stacks were recorded via a confocal microscope equipped with an Airyscan mode (CD7 Cell Discoverer with an LSM900/AiryScan module, Zeiss). Maximum projections were generated. All fluorescence pictures taken with a Zeiss microscope were processed using the Zen Blue software or ImageJ.

## Supplementary Information

Supplementary Information.
