# Supplementary material for: Patient-derived head and neck tumor slice cultures: a versatile tool to study oncolytic virus action
Source: Sci Rep. 2022 Sep 12;12:15334. doi: 10.1038/s41598-022-19555-0 (PMC9467994; doi:10.1038/s41598-022-19555-0)
Supplement: Supplementary file 1 — Supplementary Information. [file 41598_2022_19555_MOESM1_ESM.pdf]

Patient-derived head and neck tumor slice cultures-
a versatile tool to study oncolytic virus action

Authors

Annette Runge[1*], Melissa Mayr[2*], Theresa Schwaiger[2], Susanne Sprung[3], Paolo Chetta[4], Timo Gottfried[1], Jozsef Dudas[1], Maria C. Greier[1], Marlies C. Glatz[2], Christoph Schatz[3] Johannes Haybaeck[3,5], Knut Elbers[2], Herbert Riechelmann[1#], Patrik Erlmann[2#], Monika Petersson[2#+]

Affiliations

*authors contributed equally

[#] corresponding authors

[+]correspondence should be addressed to:

monika.petersson@boehringer-ingelheim.com

[1] Department of Otorhinolaryngology, Head and Neck Surgery, Medical University of Innsbruck, Innsbruck, Austria

[2] ViraTherapeutics GmbH, Rum, Austria

[3] Institute of Pathology, Neuropathology and Molecular Pathology, Medical University of Innsbruck, Innsbruck, Austria

[4] Boehringer Ingelheim RCV GmbH & Co KG, Vienna, Austria

[5] Diagnostic & Research Center for Molecular BioMedicine, Institute of Pathology, Medical University Graz, Graz, Austria

Supplementary FigureS1

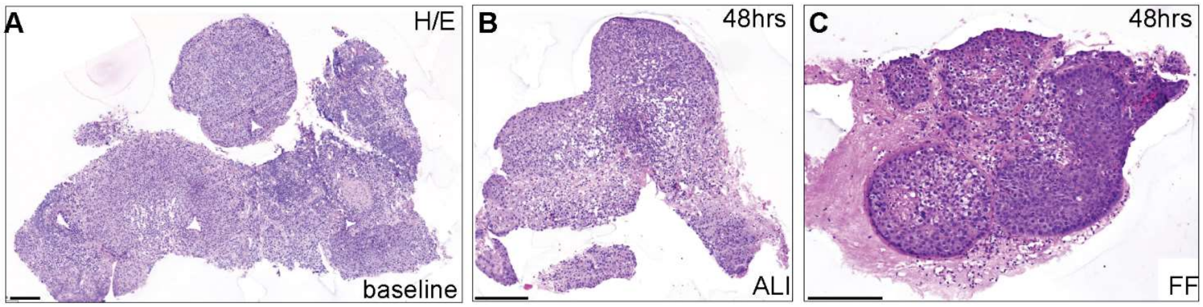

D

	Tumor (%)	Stroma (%)	Necrosis (%)
BL SC-079	90	10	20
ALI SC-079	90	10	30
FF SC-079	100	0	30
BL SC-069	80	20	0
ALI SC-069	100	0	10
FF SC-069	100	0	5

SupplFigS1 Morphology of patient-derived HNSCC slices is retained following culturing.
A-C H/E staining of patient-derived HNSCC slices at baseline, under air-liquid (ALI) or free-floating (FF) conditions. Slices were cultured for 48hrs. Scale bars represent 200µm.
D Histopathological assessment of tumor, stroma content and necrosis at baseline (BL) and ALI or FF cultures (48hrs). Examples of two cases are displayed showing that the tumor and stromal compartment is retained following cultivation with no major alterations.

Supplementary Figure2

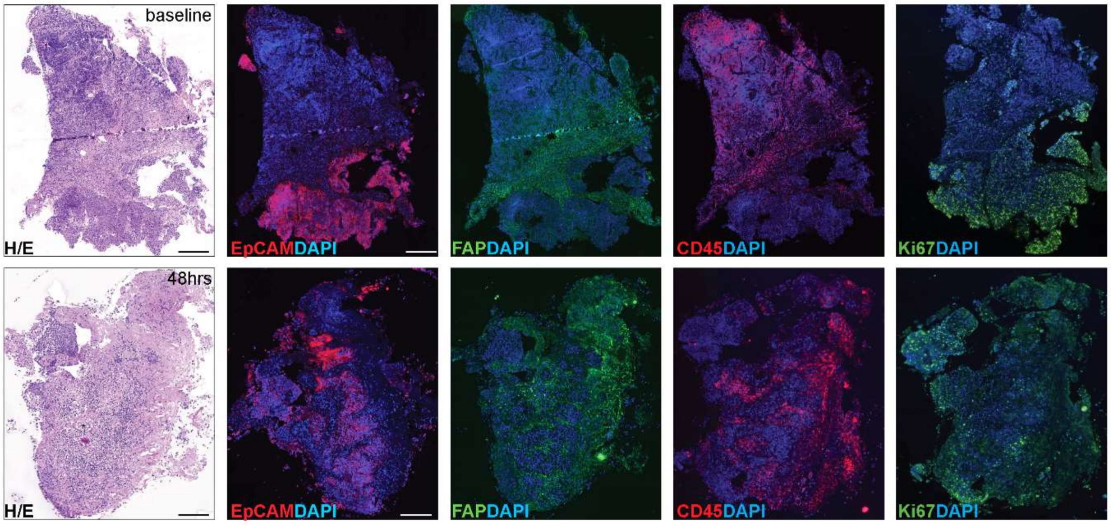

SupplFigS2 Patient-derived HNSCC slice cultures retain tissue composition ex vivo.
A,F Exemplary morphology of a second human HNSCC case (SC-052) at baseline (0hrs) and 48hrs cultured (H/E). Immunofluorescent stainings were performed on baseline (**B-E**) and 48hrs cultures (**G-J**) to analyze the presence of the tumor tissue (**B,G**) (EpCAM-red), the stromal compartment (**C,H**) (FAP-green) and the immune compartment (**D,I**) (CD45-red). Proliferation was assessed in baseline and 48hrs cultures (**E,J**) (Ki67-green). DAPI (blue) was used as nuclear counterstain. Scale bars represent 200µm.

Supplementary Figure3

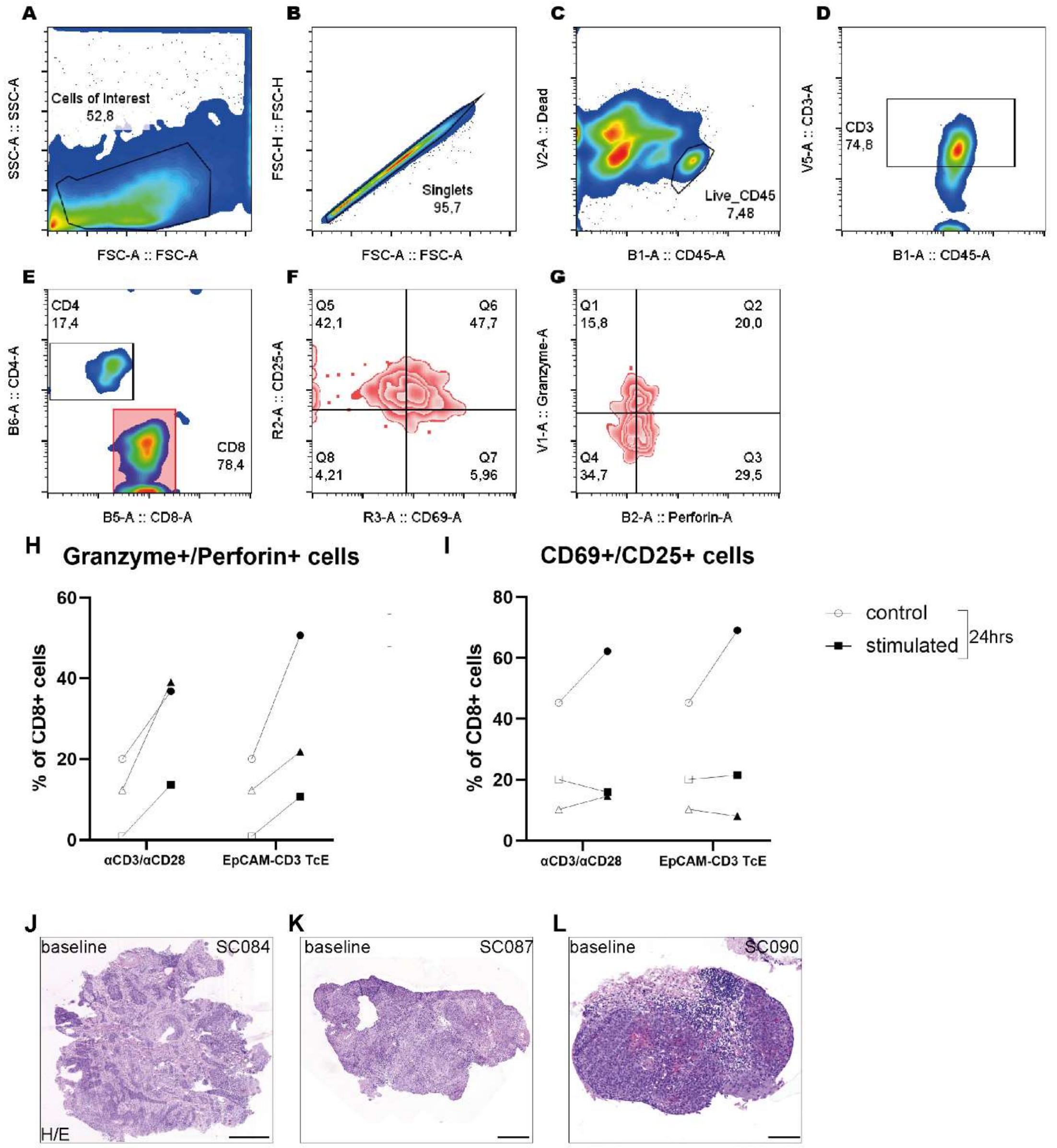

M

	SC-084	SC-087	SC-090
Tumor (%)	90	90	90
Stroma (%)	10	10	10
Necrosis (%)	5	5	0

SupplFigS3: Immune cells are functional in patient-derived HNSCC slice cultures.
After exclusion of debris and gating cells of interest (**A**), doublets were excluded from the analysis (**B**). From the remaining singlet population, living CD45^{high} cells – representing the leukocyte population – were determined (**C**). The subsequently gated CD3+ve subpopulation (**D**) was further divided into CD4 and CD8 expressing cells (**E**). From these, the frequency of CD25 and/or CD69 (**F**) as well as Granzyme B and/or Perforin expressing cells (**G**) were determined (example shown for the CD8+ve subpopulation of SC-084). Frequencies from all populations were visualized using GraphPad Prism. (**H,I**) Flow cytometric analysis of T-cell (CD8) effector molecules (Granzyme B, Perforin) and activation molecules (CD25, CD69) were performed (n=3 HNSCCs, circle-SC084, rectangle-SC087, triangle-SC090).

Samples were analyzed at 24hrs post treatment and technical triplicates were pooled. Plotted are open symbols for controls and filled symbols for stimulated. In comparison to the isotype-treated controls, an increase in frequency of CD8 cells expressing Perforin and/or Granzyme B was observed upon stimulation with α-CD3/α-CD28 or the EpCAM-CD3 TcE (**H**). Frequency of CD69 and/or CD25 expressing CD8 cells was increased in two of the three samples after stimulation (**I**). **J-M** H/E staining and histopathological assessment of three baseline HNSCC samples (**J**-SC-084, **K**-SC-087, **L**-SC-090) displaying a tumor content of 90%,10% stromal content and less than 5% necrosis. Scale bars represent 500µm for J,L and 100µm for K.

Supplementary Figure4

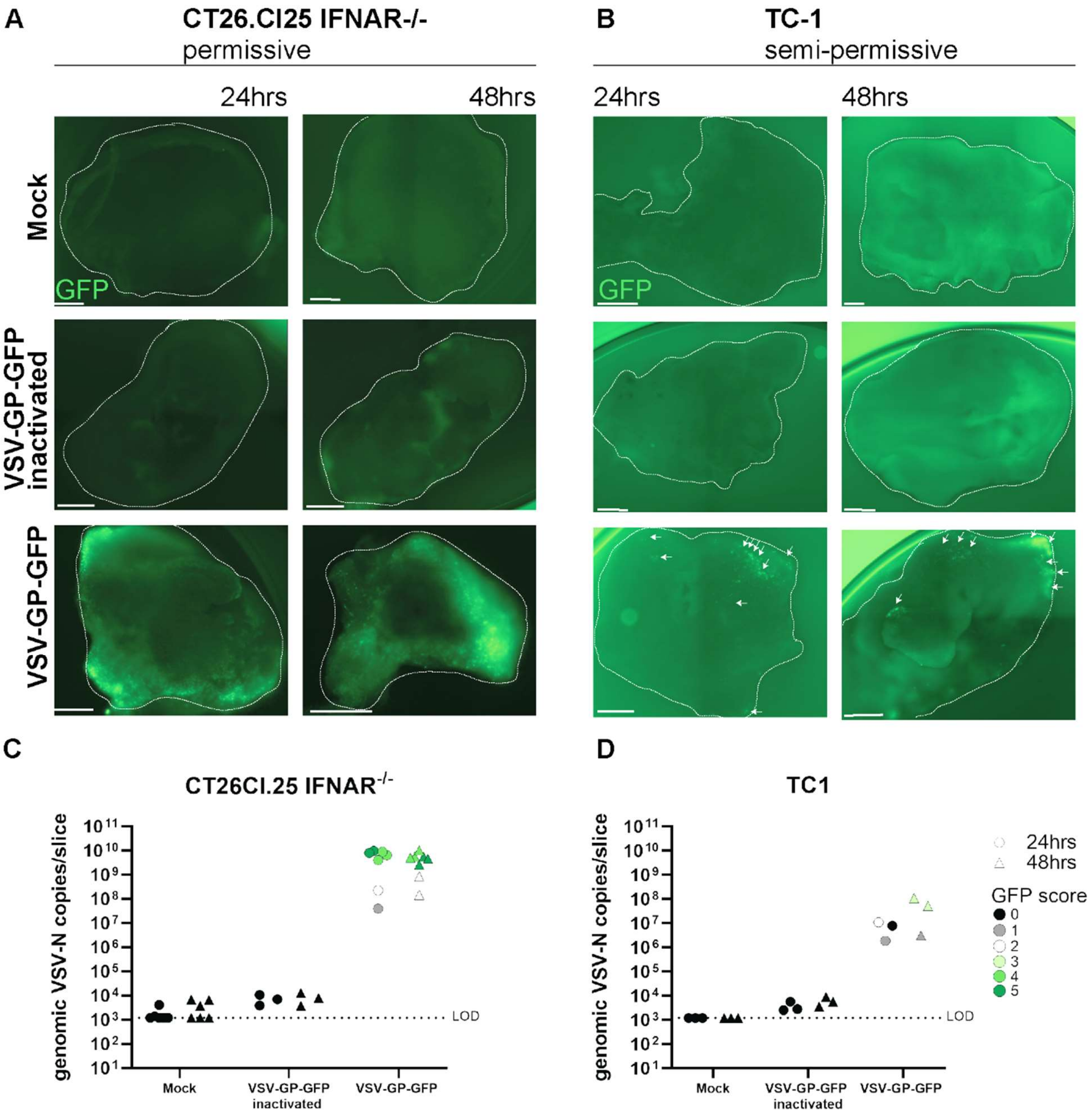

SupplFigS4: Permissivity assay was established using murine tumor slice cultures.
Slice cultures of two different murine tumor models, CT26.Cl25 IFNAR$^{-/-}$ (**A**) and TC1 (**B**) were infected with VSV-GP-GFP ex vivo and viral spread was monitored over time (24hrs and 48hrs). For both tumor models mock treated slices served as a control. UV-inactivated VSV-GP-GFP was included as control for non-replicative input virus. Live microscopy revealed an increase in GFP+ve cells peaking at 48hrs in the OV treated CT26.Cl25 IFNAR$^{-/-}$ derived slice cultures (GFP score ranged from 2-5). In contrast, small GFP+ve patches were detected (GFP score ranged from 0-3) in OV-treated TC1 derived slices. No signal was detected in the controls (**A,B**). Scale bars represent 500µm.
Murine tumor slice cultures were harvested at 24hpi or 48hpi and genomic VSV-N copy number was determined (**C,D**). Highly permissive CT26.Cl25 IFNAR$^{-/-}$ slices already showed a saturation in VSV-N copy number after 24hpi (**C**) whereas in the semi-permissive TC1 model an increase after 48hpi could be observed (**D**). The color indicates the corresponding GFP score during permissivity testing. Genomic copy numbers were measured in technical triplicates with biological triplicates for TC1 slices and inactivated virus CT26.Cl25 IFNAR$^{-/-}$ group, and biological sixtuplicates for Mock and VSV-GP-GFP treated CT26.Cl25 IFNAR$^{-/-}$ slices.

Supplementary Table1

SupplTableS1: Clinical parameters and histopathological assessment of baseline and cultured HNSCCs (n=12).
Table1 summarizes the clinical data (patient ID, gender, age, localization and pT (primary tumor) of the obtained biospecimens). For histopathological assessment, all selected cases (n=12) were invasive squamous cell carcinomas.

Patient ID	Gender	Age	Localisation	pT	Description
SC-40	female	67	Oropharynx	T1	Invasive squamous cell carcinoma
SC-41	male	75	Oropharynx	T2	Invasive squamous cell carcinoma
SC-42	male	63	Oropharynx	T2	Invasive squamous cell carcinoma
SC-46	male	77	Hypopharynx	T3	Invasive squamous cell carcinoma
SC-47	male	75	Larynx	T4a	Invasive squamous cell carcinoma
SC-20	male	51	Oropharynx	T4a	Invasive squamous cell carcinoma
SC-24	male	75	Larynx	T4a	Invasive squamous cell carcinoma
SC-13	male	45	Oropharynx	T2	Invasive squamous cell carcinoma
SC-15	male	73	Larynx	T3	Invasive squamous cell carcinoma
SC-16	male	71	Hypopharynx	T3	Invasive squamous cell carcinoma

Supplementary Table2

SupplTableS2: VSV-GP-GFP can infect different cell lineages in patient-derived HNSCC ecosystems.

Immunofluorescent studies were performed on thin sections derived from VSV-GP-GFP infected HNSCC slices harvested at 48hpi (n=11 patients, technical duplicates). Localization of the VSV Nucleoprotein (VSV-N) in indicated compartments was analyzed. While EpCAM was used as a counterstain for tumor epithelial cells, Vimentin was used to co-stain tumorous stromal cells. The table shows if epithelial tumor cells or tumorous stromal cells were present (checkmark) in the analyzed slice and if the VSV-N signal (=infected) was detected in the particular compartment (checkmark). All analyzed cultured cases (11/11) comprised tumor epithelium and 10/11 cases showed an infection. 9/11 cultured cases still retained the tumorous stromal cells; 7/9 could be infected with VSV-GP-GFP.

	Epithelial tumor cell		Tumorous stromal cells	
ID	present	infected	present	infected
SC-024	√	√	√	-
SC-040	√	√	√	√
SC-041	√	√	√	√
SC-042	√	√	√	√
SC-044	√	√	√	√
SC-045	√	√	√	√
SC-046	√	√	√	-
SC-047	√	√	√	√
SC-048	√	-	-	-
SC-049	√	√	-	-
SC-050	√	√	√	√

SupplTableS3 Primary antibodies used for immunofluorescent studies

Antibody	Reactivity	Supplier	Catalog No.	Clone	Host species	Dilution
CD45	human	Invitrogen	14-0459-82	HI30	Mouse IgG1	1:250
EpCAM	human	Abcam	ab124825	EPR677 (2)	Rabbit	1:200
FAPα	human	Abcam	ab207178	EPR20021	Rabbit	1:100
Ki67	human	Abcam	ab15580	polyclonal	Rabbit	1:250
Vimentin	human	Abcam	ab92547	EPR3776	Rabbit	1:1000
aCas3	human	Cell signaling	9661	polyclonal	Rabbit	1:200
VSV-N	VSV- Ind nucleo-capsid (N) protein	Kerafast	EB0009	10G4	Mouse IgG2a	1:250

SupplTableS4 Secondary antibodies used for immunofluorescent studies

Antibody/reactivity/fluorochrome	Supplier	Catalog No.	Dilution
Goat – α-MouseIgG1 Alexa Fluor 647	ThermoFisher	A-21240	1:750
Goat – α-MouseIgG2a Alexa Fluor 568	ThermoFisher	A-21134	1:750
Goat – α-MouseIgG2a Alexa Fluor 594	ThermoFisher	A-21135	1:750
Goat – α-Rabbit Alexa Fluor 568	ThermoFisher	A-11036	1:750
Goat – α-Rabbit Alexa Fluor 594	ThermoFisher	A-11012	1:750
Goat – α-Rabbit Alexa Fluor 647	ThermoFisher	A-21245	1:750
